# A Two-Stage Transformer Framework for Sparse-Array Direction-of-Arrival Estimation via Correlation Vector Recovery

**DOI:** 10.3390/s26103132

**Published:** 2026-05-15

**Authors:** Wenchao He, Yiran Shi, Hongxi Zhao, Hongliang Zhu, Chunshan Bao

**Affiliations:** 1 School of Mechanical and Electrical Engineering, Changchun Humanities and Sciences College, Changchun 130117, China; hewc23@mails.jlu.edu.cn; 2 College of Communications Engineering, Jilin University, Changchun 130015, China; hxzhao24@mails.jlu.edu.cn (H.Z.); zhuhl24@mails.jlu.edu.cn (H.Z.); baocs24@mails.jlu.edu.cn (C.B.)

**Keywords:** direction-of-arrival estimation, sparse array, transformer, covariance completion

## Abstract

Accurate direction-of-arrival (DOA) estimation with high resolution is fundamental to many array sensing applications. In practice, however, sparse arrays with missing sensors and snapshot-limited observations often lead to incomplete and noisy second-order statistics, which substantially degrades the performance of conventional eigendecomposition-based estimators. In this paper, we propose a two-stage Transformer framework for sparse-array DOA estimation that explicitly separates correlation recovery from angle inference. The first stage operates in the correlation domain and learns to reconstruct a clean and complete correlation vector from partially observed measurements using masking-aware tokenization and global-context modeling. The recovered representation can be further converted into a structured covariance matrix, providing an interpretable interface to classical signal processing back-ends. Based on the recovered features, the second stage adopts a Transformer regressor to directly predict multi-source DOAs. Extensive simulations on a large-scale dataset with SNRs from −5 to 10 dB and various snapshot numbers demonstrate that the proposed method delivers robust accuracy and improved stability in low-SNR and snapshot-limited regimes, while maintaining competitive performance at higher SNRs. Additional evaluations with an ESPRIT back-end further confirm that the recovery-based covariance yields more reliable DOA estimation than conventional difference–coarray processing, with particularly evident gains under challenging noise conditions.

## 1. Introduction

Direction-of-arrival (DOA) estimation is a core task in array signal processing with broad applications in radar, sonar, wireless communications, and acoustic localization. Given multi-sensor observations, the goal is to infer the incident angles of multiple far-field sources [1,2]. Over the past decades, a rich family of classical estimators has been developed, among which subspace-based techniques such as MUSIC [3] and ESPRIT [4] remain particularly popular due to their high resolution and clear physical interpretation [5,6,7].

Despite their success under ideal conditions, conventional DOA estimators often rely on accurate second-order statistics. In practical deployments, this prerequisite is frequently violated. For example, physical constraints, sensor failures, or cost limitations may lead to sparse arrays with missing elements, where only a subset of candidate sensor locations can be observed. When sensors are missing, the sample covariance becomes incomplete; naive zero-filling introduces structured bias, and under a limited number of snapshots the covariance estimate can be noisy or ill-conditioned, which directly degrades eigen decomposition-based DOA pipelines [8,9,10,11].

Sparse-array processing methods based on virtual arrays and difference coarrays provide another classical route to extend the effective aperture, while powerful, these approaches typically depend on reliable covariance estimation and often require additional procedures such as completion, smoothing, or other refinements when facing sensor holes and limited snapshots [12,13]. As a result, their performance can be sensitive to mismatch, and the overall processing chain may become increasingly complex in challenging environments [14,15,16].

Motivated by these limitations, learning-based DOA estimation has attracted growing attention in recent years. Neural networks can learn robust mappings from data to DOA parameters and can potentially reduce the reliance on carefully engineered intermediate steps. For instance, Liu et al. developed a CNN-based DOA estimator for underwater acoustic arrays by feeding covariance-related representations into a convolutional network, demonstrating improved performance compared with conventional pipelines in their considered scenarios [17]. Nevertheless, such covariance-matrix-driven CNN approaches typically treat the input as a regular grid and rely on a complete and reliable covariance estimate, which may become problematic for sparse arrays with missing sensors and for snapshot-limited measurements where the covariance is incomplete or severely distorted. In contrast, our method explicitly addresses this bottleneck by introducing a dedicated correlation-domain recovery stage that reconstructs a clean and complete correlation vector before DOA inference, thereby providing a more stable and physically consistent representation for subsequent estimation under challenging low-SNR and limited-snapshot conditions [18,19,20].

More recently, Transformer-based architectures have been introduced into DOA estimation due to their ability to model global dependencies and long-range contextual relationships [21]. Compared with convolutional networks, which mainly extract local patterns from covariance or spectrum-like inputs, the self-attention mechanism can adaptively capture interactions among different sensors, snapshots, or feature tokens [22]. Existing Transformer-based DOA methods generally employ the Transformer as a feature extractor or regression module for direct angle estimation, where the input is usually constructed from snapshots, covariance matrices, or spectrum-like representations, and the network directly outputs the estimated DOAs. These studies demonstrate the potential of attention-based modeling for improving DOA estimation performance [23].

However, existing Transformer-based DOA estimators are mainly designed for the final DOA inference stage. In these methods, the Transformer is typically used to extract global features from snapshots, covariance matrices, or spectrum-like representations, and the estimated DOAs are directly obtained from the learned features. For sparse-array scenarios with missing or inactive sensors, however, the difficulty is not limited to angular regression. The incomplete spatial sampling also leads to missing or unreliable correlation-domain information, which can further degrade the subsequent DOA estimation performance. Motivated by this observation, the proposed method introduces a recovery-before-inference strategy. Specifically, the first-stage Transformer performs mask-aware correlation-vector recovery to reconstruct a clean and complete reference correlation vector from partial sparse-array observations, while the second-stage Transformer conducts multi-source DOA regression based on the recovered representation.

In this work, we propose a two-stage Transformer framework for sparse-array DOA estimation that explicitly separates “recovery” from “inference”. Instead of directly operating on the full covariance matrix, we adopt a compact correlation-domain representation anchored at a reference sensor, which can be interpreted as the first column of the covariance matrix. The first stage learns to reconstruct a clean and complete correlation vector from partially observed measurements by leveraging masking-aware token design and Transformer-based global context modeling. This recovered representation can serve as an interpretable bridge to conventional second-order processing and provides a more reliable input for downstream DOA estimation.

Building on the recovered representation, the second stage employs a Transformer regressor to map the per sensor recovered features to multi-source DOA estimates. By combining correlation-domain completion with data-driven regression, the proposed framework aims to achieve stable performance across a wide range of SNRs and snapshot numbers, while remaining effective under sensor holes and snapshot-limited conditions.

It should be emphasized that the major advance of the proposed framework does not lie in simply applying a Transformer to DOA regression. Existing deep-learning-based DOA estimators, including several Transformer-related schemes, mainly learn a direct mapping from snapshots, covariance matrices, or spectrum-like features to angular parameters. In these methods, the Transformer is generally used as a feature extractor or regression module for the final DOA inference stage. In contrast, sparse-array DOA estimation with missing or inactive sensors involves an additional difficulty: the available observations provide only incomplete and noisy correlation-domain information. Therefore, directly mapping the degraded observations to DOAs may limit the interpretability and robustness of the estimation process. Motivated by this observation, the proposed method explicitly separates correlation-domain recovery from DOA inference and introduces a recovery-before-inference paradigm for sparse-array DOA estimation.

The main contributions and novelties of this work are summarized in three aspects. First, a two-stage Transformer framework is developed for sparse-array DOA estimation, where mask-aware correlation-vector recovery is performed before multi-source angle inference. This design shifts the role of Transformer modeling from only final DOA regression to the reconstruction of reliable correlation-domain information from partial sparse-array observations. Second, the recovered reference correlation vector is physically interpretable because it corresponds to the first column of the covariance matrix. Under the ULA structural constraint, it can be further lifted to a Hermitian Toeplitz covariance matrix, thereby building an interpretable bridge between learning-based recovery and classical covariance-domain DOA processing. Third, comprehensive experiments are conducted on a large-scale dataset generated from a six-sensor sparse array configuration under different SNR levels and snapshot numbers. Comparisons with representative classical algorithms and learning-based baselines demonstrate that the proposed method achieves more robust and accurate DOA estimation in low- and medium-SNR regimes, where covariance estimation is more seriously affected by noise and sparse-array observations. In addition, the ESPRIT back-end verification confirms that the recovered vector is not merely a latent neural feature but a physically meaningful covariance-domain representation compatible with classical DOA processing. The array model perturbation experiment further shows that the proposed method maintains a certain degree of robustness against practical sensor-position errors.

## 2. Methods

### 2.1. Signal Model

Consider a linear array with *N* candidate sensor locations. Due to physical constraints, only a subset of sensors is available. Let

(1) O⊂{0,1,…,N−1}

denote the index set of observed sensors with |O|<N and assume the reference sensor is observed:

(2) 0∈O.

Assume *M* far-field narrowband sources array. The received complex envelope at sensor *n* and snapshot *t* is

(3) $$x_{n}\left(t\right)=\sum_{m=1}^{M}a_{n}\left(\theta_{m}\right)s_{m}\left(t\right)+v_{n}\left(t\right)$$

where $s_{m}\left(t\right)\in C$ is the waveform of the *m*th source, $v_{n}\left(t\right)\in C$ is additive noise, and $\theta_{m}$ is the DOA of the *m*th source.

Stacking all sensors yields the snapshot vector

(4) $$x\left(t\right)={\left[x_{0}\left(t\right),x_{1}\left(t\right),\ldots ,x_{N-1}\left(t\right)\right]}^{T}$$

and the compact model

(5) x(t)=A(θ)s(t)+v(t)

where $s\left(t\right)={\left[s_{1}\left(t\right),\ldots ,s_{M}\left(t\right)\right]}^{T}$ and $v\left(t\right)={\left[v_{0}\left(t\right),\ldots ,v_{N-1}\left(t\right)\right]}^{T}$.

For an ULA with spacing d=λ/2, the steering vector is

(6) $$a\left(\theta \right)={\left[1,e^{-j2\pi d\sin\theta /\lambda },\ldots ,e^{-j2\pi (N-1)d\sin\theta /\lambda }\right]}^{T}$$

and the manifold matrix is

(7) $$A\left(\theta \right)=\left[a\left(\theta_{1}\right),\ldots ,a\left(\theta_{M}\right)\right].$$

A central second-order statistic is the covariance matrix

(8) $$R=E\left[x\left(t\right)x^{H}\left(t\right)\right]$$

with the sample estimate

(9) $$\hat{R}=\frac{1}{T}\sum_{t=1}^{T}x\left(t\right)x^{H}\left(t\right).$$

When sensors are missing, x(t) is only partially observed, so $\hat{R}$ is effectively missing rows and columns. Naively zero-filling unobserved channels introduces structured bias. Moreover, for limited snapshots *T*, $\hat{R}$ becomes noisy and can be rank-deficient, which degrades subspace-based DOA estimators.

### 2.2. Problem Formulation and Overview of the Proposed Framework

Based on the signal model above, the problem considered in this paper can be stated as follows. Given the partially observed sparse-array snapshots ${\left\{x_{O}\left(t\right)\right\}}_{t=1}^{T}$, where O denotes the set of available sensors, the objective is to estimate the multi-source DOA vector $\theta ={\left[\theta_{1},\theta_{2},\ldots ,\theta_{M}\right]}^{T}$. Sparse arrays can increase the effective aperture through virtual-array or difference-coarray processing. However, such methods still rely on the quality of the sample covariance or the derived coarray correlation vector. In low-SNR and limited-snapshot regimes, the estimated covariance is noisy and may be ill-conditioned, and the virtual-array statistics obtained from difference coarrays can be affected by estimation errors, missing lags, and smoothing-related degradation. Therefore, the central problem addressed in this work is not merely the existence of sparse physical sensors, but how to obtain a reliable and complete correlation-domain representation from degraded sparse-array observations.

Compared with conventional difference–coarray processing, the proposed framework does not directly treat the noisy sample covariance as the final statistic for virtual-array DOA estimation. Instead, it decomposes sparse-array DOA estimation into two consecutive tasks: correlation-domain recovery and DOA inference. In the first stage, a mask-aware Transformer is used to recover a clean and complete reference correlation vector from partial and noisy correlation measurements. The observation mask and sensor-position information are explicitly encoded into the input tokens, so that unavailable entries are treated as missing observations rather than true zero-valued measurements. This enables the model to learn global dependencies among observed and unobserved correlation entries and to suppress part of the noise-induced distortion before DOA estimation.

In the second stage, the recovered correlation-domain representation is used as the input of another Transformer regressor to estimate the multi-source DOAs. In addition, because the recovered reference correlation vector corresponds to the first column of the covariance matrix, it can be lifted to a Hermitian Toeplitz covariance matrix under the ULA structural constraint and further used by classical subspace-style estimators such as ESPRIT. Therefore, the proposed method differs from conventional difference–coarray processing in that it introduces a learnable recovery step before angle inference, aiming to provide a more reliable second-order representation under low-SNR, limited-snapshot, and sparse-observation conditions. The detailed construction of the correlation-domain input, the Transformer-based recovery module, the covariance reconstruction procedure, and the final DOA regression module are presented in the following subsections.

**Figure 1:**
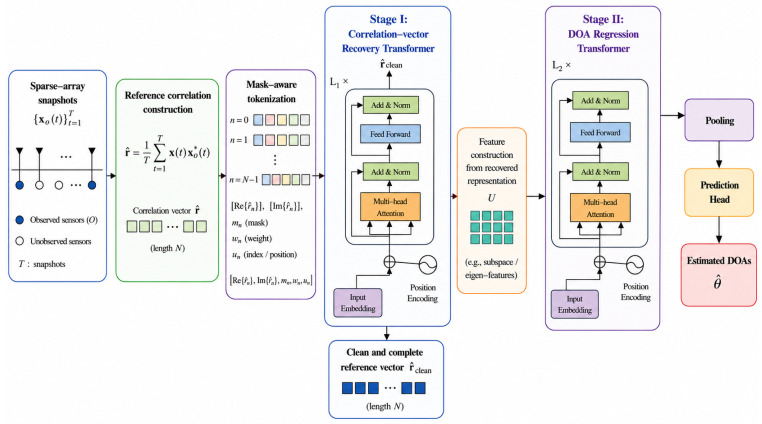
Flowchart of the proposed method.

The overall workflow of the proposed method is illustrated in Figure 1, and the detailed technical modules are elaborated in the following subsections.

### 2.3. Data Construction

Instead of working with the full covariance matrix, we adopt a compact correlation-domain statistic anchored at the reference sensor. Define the reference cross-correlation vector

(10) $$r=E\left[x\left(t\right)x_{0}^{*}\left(t\right)\right]$$

and its sample estimate

(11) $$\hat{r}=\frac{1}{T}\sum_{t=1}^{T}x\left(t\right)x_{0}^{*}\left(t\right).$$

Let $e_{0}\in R^{N}$ denote the first canonical basis vector. By definition of x(t), the reference-sensor snapshot satisfies

(12) $$x_{0}\left(t\right)=e_{0}^{T}x\left(t\right).$$

Taking complex conjugation and using ${\left(e_{0}^{T}x\left(t\right)\right)}^{*}=x^{H}\left(t\right)e_{0}$ yields

(13) $$x_{0}^{*}\left(t\right)=x^{H}\left(t\right)e_{0}.$$

Substituting (13) into (10) gives

(14) $$r=E\left[x\left(t\right)x_{0}^{*}\left(t\right)\right]=E\left[x\left(t\right)x^{H}\left(t\right)\right]e_{0}.$$

Since $R=E\left[x\left(t\right)x^{H}\left(t\right)\right]$, we obtain

(15) $$r=Re_{0}$$

which shows that r is exactly the first column of the covariance matrix R.

This is precisely why a recovered r can be used to reconstruct R under appropriate structural assumptions, as detailed in Section 2.6.

Because only sensors in O are available, the entries ${\hat{r}}_{n}$ are missing for n∉O. In our data organization, we set

(16) $${\hat{r}}_{n}=0\;\forall n\notin O$$

and provide an explicit observation indicator to the network so that zeros at holes are interpreted as masked tokens rather than true measurements.

### 2.4. Conventional Virtual-Array Formulation for Sparse Arrays

We briefly revisit the conventional difference-coarray route to highlight what our method avoids. Under the standard assumptions of uncorrelated sources and independent noise, the covariance can be written as

(17) $$R=A\left(\theta \right)PA^{H}\left(\theta \right)+\sigma^{2}I_{N}$$

where $P=\mathrm{diag}(p_{1},\ldots ,p_{M})$.

Vectorizing R yields

(18) z=vec(R).

Using $\mathrm{vec}\left(UVW\right)=\left(W^{T}⊗U\right)\mathrm{vec}\left(V\right)$, we obtain

(19) $$z=\left(A^{*}⊙A\right)p+\sigma^{2}\mathrm{vec}\left(I_{N}\right)$$

where ⊙ is the Khatri–Rao product and $p={\left[p_{1},\ldots ,p_{M}\right]}^{T}$.

The virtual manifold columns are

(20) $$a_{v}\left(\theta \right)=a^{*}\left(\theta \right)⊗a\left(\theta \right).$$

whose entries depend on sensor index differences, thus forming a difference coarray, while powerful, this approach typically requires accurate $\hat{R}$ and involves higher-dimensional processing. Under sensor holes and limited snapshots, covariance completion and smoothing are often needed, increasing complexity and error sensitivity.

### 2.5. Signal Recovery Based on Transformer

Our first stage learns a mapping from the partially observed and noisy $\hat{r}$ to its clean and complete counterpart r. Importantly, the network outputs a signal vector in the correlation domain, namely the full-length complex vector corresponding to the first column of the covariance matrix.

#### 2.5.1. Input Token Construction

We build a length-*N* token sequence. For each sensor index *n*, we define a 5-dimensional real-valued token

(21) $$x_{n}=\left[x_{n}^{\left(1\right)},x_{n}^{\left(2\right)},x_{n}^{\left(3\right)},x_{n}^{\left(4\right)},x_{n}^{\left(5\right)}\right]$$

with the following components.

- Complex observation split into real and imaginary parts:
  (22) $$x_{n}^{\left(1\right)}=ℜ\left({\hat{r}}_{n}\right)$$
  (23) $$x_{n}^{\left(2\right)}=ℑ\left({\hat{r}}_{n}\right).$$
- Observation indicator:
  (24) $$x_{n}^{\left(3\right)}=m_{n}$$
  where $m_{n}=1$ if n∈O, and $m_{n}=0$ otherwise.
- Observation weight:
  (25) $$x_{n}^{\left(4\right)}=w_{n}$$
  where $w_{n}=1/\left|O\right|$ if n∈O, and $w_{n}=0$ otherwise.
- Normalized sensor index:
  (26) $$x_{n}^{\left(5\right)}=u_{n}$$
  with
  (27) $$u_{n}=\frac{2n}{N-1}-1.$$

Stacking all tokens yields the input feature matrix

(28) $$X={\left[x_{0}^{T},x_{1}^{T},\ldots ,x_{N-1}^{T}\right]}^{T}.$$

The supervision target is the clean complex vector r split into two channels:

(29) $$Y=\left[ℜ(r),ℑ(r)\right]\in R^{N\times 2}.$$

#### 2.5.2. Normalization Strategy

To stabilize training, we standardize real and imaginary parts using training-set statistics computed from clean targets at observed sensors. Let $\left(\mu_{\mathrm{re}},\sigma_{\mathrm{re}}\right)$ and $\left(\mu_{\mathrm{im}},\sigma_{\mathrm{im}}\right)$ denote these statistics. For the input channels, only observed entries are standardized, while holes remain 0:

(30) $$ℜ\left({\hat{r}}_{n}\right)\leftarrow \left\{\begin{array}{cc} \frac{ℜ\left({\hat{r}}_{n}\right)-\mu_{\mathrm{re}}}{\sigma_{\mathrm{re}}} & m_{n}=1\\ 0 & m_{n}=0 \end{array}\right.$$

(31) $$ℑ\left({\hat{r}}_{n}\right)\leftarrow \left\{\begin{array}{cc} \frac{ℑ\left({\hat{r}}_{n}\right)-\mu_{\mathrm{im}}}{\sigma_{\mathrm{im}}} & m_{n}=1\\ 0 & m_{n}=0. \end{array}\right.$$

For the targets, all entries are standardized:

(32) $$ℜ\left(r_{n}\right)\leftarrow \frac{ℜ\left(r_{n}\right)-\mu_{\mathrm{re}}}{\sigma_{\mathrm{re}}}$$

(33) $$ℑ\left(r_{n}\right)\leftarrow \frac{ℑ\left(r_{n}\right)-\mu_{\mathrm{im}}}{\sigma_{\mathrm{im}}}$$

where $r_{n}$ denotes the *n*th entry of r. This asymmetry ensures that zeros at holes remain a consistent mask token at the network input, while the network is still supervised to predict valid values at holes.

#### 2.5.3. Transformer Encoder: Embedding and Positional Encoding

Each token $x_{n}\in R^{5}$ is linearly embedded into a *d*-dimensional vector:

(34) $$e_{n}=W_{\mathrm{emb}}x_{n}+b_{\mathrm{emb}}$$

where $W_{\mathrm{emb}}\in R^{d\times 5}$ and $b_{\mathrm{emb}}\in R^{d}$.

Let $E\in R^{N\times d}$ be the stacked embedding matrix. We add a sinusoidal positional encoding $F\in R^{N\times d}$ with

(35) $$F_{n,2k}=\sin\left(\frac{n}{\omega_{k}}\right)$$

(36) $$F_{n,2k+1}=\cos\left(\frac{n}{\omega_{k}}\right)$$

(37) $$\omega_{k}=10000^{2k/d}$$

and obtain the Transformer input

(38) $$H^{\left(0\right)}=E+F.$$

#### 2.5.4. Attention Model

At layer *ℓ*, given token features $H^{\left(\ell -1\right)}\in R^{N\times d}$, we form queries, keys, and values by linear projections

(39) $$Q^{\left(\ell \right)}=H^{\left(\ell -1\right)}W_{Q}^{\left(\ell \right)}$$

(40) $$K^{\left(\ell \right)}=H^{\left(\ell -1\right)}W_{K}^{\left(\ell \right)}$$

(41) $$V^{\left(\ell \right)}=H^{\left(\ell -1\right)}W_{V}^{\left(\ell \right)}$$

where $W_{Q}^{\left(\ell \right)},W_{K}^{\left(\ell \right)},W_{V}^{\left(\ell \right)}\in R^{d\times d}$.

The scaled dot-product attention score matrix is

(42) $$S^{\left(\ell \right)}=\frac{Q^{\left(\ell \right)}{\left(K^{\left(\ell \right)}\right)}^{T}}{\sqrt{d}}$$

and the attention weight matrix is obtained by row-wise softmax

(43) $${\mathrm{Attention}}^{\left(\ell \right)}=\mathrm{Softmax}\left(S^{\left(\ell \right)}\right)$$

where ${\mathrm{Attention}}^{\left(\ell \right)}\in R^{N\times N}$ and each row sums to one.

The attention output is

(44) $$Z^{\left(\ell \right)}={\mathrm{Attention}}^{\left(\ell \right)}V^{\left(\ell \right)}.$$

#### 2.5.5. Multi-Head Self-Attention Expanded

In multi-head attention with *G* heads, we split the embedding dimension into $d_{g}=d/G$. For head *g*, we use head-specific projections

(45) $$Q_{g}^{\left(\ell \right)}=H^{\left(\ell -1\right)}W_{Q,g}^{\left(\ell \right)}$$

(46) $$K_{g}^{\left(\ell \right)}=H^{\left(\ell -1\right)}W_{K,g}^{\left(\ell \right)}$$

(47) $$V_{g}^{\left(\ell \right)}=H^{\left(\ell -1\right)}W_{V,g}^{\left(\ell \right)}$$

where $W_{Q,g}^{\left(\ell \right)},W_{K,g}^{\left(\ell \right)},W_{V,g}^{\left(\ell \right)}\in R^{d\times d_{g}}$.

The head output is

(48) $$Z_{g}^{\left(\ell \right)}=\mathrm{Softmax}\left(\frac{Q_{g}^{\left(\ell \right)}{\left(K_{g}^{\left(\ell \right)}\right)}^{T}}{\sqrt{d_{g}}}\right)V_{g}^{\left(\ell \right)}.$$

Concatenating all heads and applying an output projection yields

(49) $$\mathrm{MHA}\left(H^{\left(\ell -1\right)}\right)=\mathrm{Concat}\left(Z_{1}^{\left(\ell \right)},\ldots ,Z_{G}^{\left(\ell \right)}\right)W_{O}^{\left(\ell \right)}$$

where $W_{O}^{\left(\ell \right)}\in R^{d\times d}$.

#### 2.5.6. Feed-Forward Network and Layer Structure

Each Transformer encoder layer consists of MHA followed by a position-wise feed-forward network with residual connections and layer normalization. Let LN(·) denote LayerNorm and let Drop(·) denote dropout. The attention sub-layer is

(50) $${\tilde{H}}^{\left(\ell \right)}=\mathrm{LN}\left(H^{\left(\ell -1\right)}+\mathrm{Drop}\left(\mathrm{MHA}\left(H^{\left(\ell -1\right)}\right)\right)\right).$$

The FFN sub-layer is

(51) $$\mathrm{FFN}\left(u\right)=W_{2}\;\phi \left(W_{1}u+b_{1}\right)+b_{2}$$

where ϕ(·) is a pointwise nonlinearity, and the layer output is

(52) $$H^{\left(\ell \right)}=\mathrm{LN}\left({\tilde{H}}^{\left(\ell \right)}+\mathrm{Drop}\left(\mathrm{FFN}\left({\tilde{H}}^{\left(\ell \right)}\right)\right)\right).$$

Stacking *L* layers produces the final token matrix

(53) $$H^{\left(L\right)}=\mathrm{Encoder}\left(H^{\left(0\right)}\right).$$

#### 2.5.7. Output Head for Complex Vector Recovery

We use a lightweight token-wise head to map each token embedding to real and imaginary outputs:

(54) $$\hat{Y}=H^{\left(L\right)}W_{\mathrm{out}}+b_{\mathrm{out}}$$

where $W_{\mathrm{out}}\in R^{d\times 2}$ and $b_{\mathrm{out}}\in R^{2}$.

The recovered complex correlation vector is then

(55) $${\hat{r}}_{n}^{\mathrm{clean}}={\hat{Y}}_{n,1}+j{\hat{Y}}_{n,2}.$$

#### 2.5.8. Loss Function with Full-Node Supervision and Diagnostics

We train using the mean squared error over all nodes in the complex plane:

(56) $$L_{\mathrm{rec}}=\frac{1}{N}\sum_{n=0}^{N-1}{\left({\hat{Y}}_{n,1}-Y_{n,1}\right)}^{2}+{\left({\hat{Y}}_{n,2}-Y_{n,2}\right)}^{2}.$$

In addition to the overall recovery error, we further report two complementary RMSE measures evaluated on the observed sensors and on the hole sensors, respectively. This separation provides a more interpretable diagnosis of the recovery behavior, because the error on O mainly reflects denoising on available measurements, whereas the error on $O^{c}$ mainly reflects interpolation on unobserved positions. Let $O^{c}$ denote the complement of O. Define

(57) $${\mathrm{RMSE}}_{\mathrm{obs}}=\sqrt{\frac{1}{\left|O\right|}\sum_{n\in O}{\left|{\hat{r}}_{n}^{\mathrm{clean}}-r_{n}\right|}^{2}}$$

(58) $${\mathrm{RMSE}}_{\mathrm{mis}}=\sqrt{\frac{1}{|O^{c}|}\sum_{n\in O^{c}}{\left|{\hat{r}}_{n}^{\mathrm{clean}}-r_{n}\right|}^{2}}.$$

#### 2.5.9. Optional Random Masking on Observed Entries

To improve robustness against varying hole patterns and sensor dropouts, we optionally apply additional random masking to observed sensors during training. Let π denote the masking probability. For observed indices, we randomly set the input real and imaginary parts to zero:

(59) $$ℜ\left({\hat{r}}_{n}\right)\leftarrow 0\;ℑ\left({\hat{r}}_{n}\right)\leftarrow 0\;\mathrm{with}\;\mathrm{probability}\;\pi \;\mathrm{for}\;n\in O.$$

The supervision remains full-node, so the model is forced to reconstruct masked observed entries from context.

### 2.6. Recovery of Covariance Matrix

From Section 2.3, $r=Re_{0}$ and therefore ${\hat{r}}^{clean}$ estimates the first column of R. If one makes no further assumptions, a single column does not uniquely determine the entire matrix. However, for a ULA under wide-sense stationarity across sensors, the covariance exhibits a Toeplitz structure, and the first column is sufficient.

Under spatial stationarity, the covariance depends only on sensor index differences:

(60) $$R_{i,j}=c_{i-j}$$

for some sequence $\left\{c_{k}\right\}$. Moreover, R is Hermitian, implying

(61) $$c_{-k}=c_{k}^{*}.$$

Let the first column be

(62) $$c={\left[c_{0},c_{1},\ldots ,c_{N-1}\right]}^{T}.$$

Then the Toeplitz covariance matrix is

(63) $$R_{T}=\mathrm{Toeplitz}\left(c,c^{*}\right)$$

where the first row is $\left[c_{0},c_{1}^{*},\ldots ,c_{N-1}^{*}\right]$.

Since r is the first column of R, we identify

(64) c=r

and thus reconstruct the covariance from the recovered vector by

(65) $${\hat{R}}_{T}=\mathrm{Toeplitz}\left({\hat{r}}^{\mathrm{clean}},{\left({\hat{r}}^{\mathrm{clean}}\right)}^{*}\right).$$

In finite snapshots, estimation noise can make ${\hat{R}}_{T}$ ill-conditioned. A standard remedy is diagonal loading:

(66) $${\hat{R}}_{L}={\hat{R}}_{T}+\epsilon I_{N}$$

where ϵ>0 is a small constant.

Once a covariance estimate is available, classical DOA estimators can be applied. For example, an eigendecomposition

(67) $${\hat{R}}_{L}=U\Lambda U^{H}$$

separates signal and noise subspaces, enabling MUSIC-type spectral search or ESPRIT-type rotational invariance methods. In our work, this reconstruction mainly serves as an interpretable bridge from the recovered signal vector to conventional second-order processing.

### 2.7. DOA Estimation Based on Transformer

After the preceding recovery stage, a complete and denoised correlation-domain vector is obtained. Instead of relying solely on a hand-crafted subspace pipeline, we further learn a direct mapping from the recovered representation to DOAs using a second-stage Transformer regressor. This stage reuses the same Transformer encoder module described above, and we only specify the stage-specific input normalization, pooling, and regression head.

For each sample, we construct a per sensor feature sequence:

(68) $$U\in R^{N\times F}$$

where *F* matches the feature dimension stored in the DOA dataset text file. The entire feature tensor is standardized using training-set statistics computed over all sensors and all training samples. Let $\mu \in R^{F}$ and $\sigma \in R^{F}$ denote these statistics. The normalized input is

(69) $${\tilde{U}}_{n,f}=\frac{U_{n,f}-\mu_{f}}{\sigma_{f}}.$$

In addition, the regressor applies a per-sample LayerNorm across feature channels before token encoding, which improves stability without changing the dataset-level normalization rule.

Feeding $\tilde{U}$ into the Transformer encoder yields token embeddings

(70) $$H=T(\tilde{U}).$$

We aggregate token embeddings into a global representation by either mean pooling or attention pooling.

Mean pooling is

(71) $$h=\frac{1}{N}\sum_{n=0}^{N-1}H_{n,:}.$$

Attention pooling uses a learnable query vector $q\in R^{d}$ to compute token weights:

(72) $$\alpha_{n}=\frac{\exp\left(q^{T}H_{n,:}/\sqrt{d}\right)}{\sum_{i=0}^{N-1}\exp\left(q^{T}H_{i,:}/\sqrt{d}\right)}$$

(73) $$h=\sum_{n=0}^{N-1}\alpha_{n}H_{n,:}.$$

If SNR metadata is available, we optionally condition the pooled feature by adding an SNR embedding:

(74) $$h\leftarrow h+\phi_{snr}\left(\mathrm{snr}\right)$$

where $\phi_{snr}\left(\cdot \right)$ is a small MLP mapping a scalar to $R^{d}$.

Finally, a lightweight regression head outputs the *K* DOAs:

(75) $$\hat{\theta }=\psi \left(h\right).$$

We minimize mean squared error between predicted and ground-truth DOAs:

(76) $$L_{\mathrm{doa}}=\frac{1}{K}{∥\hat{\theta }-\theta ∥}_{2}^{2}.$$

The detailed procedure of the proposed algorithm is summarized in Algorithm 1.

**Algorithm 1** The Proposed Sparse-Array DOA Estimation Method.**Require:** Partially observed snapshots ${\left\{x\left(t\right)\right\}}_{t=1}^{T}$ with hole set O, ground-truth DOAs θ**Ensure:** Estimated DOAs $\hat{\theta }$ (and recovered representation ${\hat{r}}_{\mathrm{clean}}$)
  1:**(Correlation construction)** Compute$\hat{r}=\frac{1}{T}\sum_{t=1}^{T}x\left(t\right)x_{0}^{*}\left(t\right),$
and apply a hole mask: ${\hat{r}}_{n}\leftarrow 0$ for n∉O.  2:**(Stage-I tokens)** Form token matrix $X\in R^{N\times 5}$ by stacking $ℜ(\hat{r})$, $ℑ(\hat{r})$, $m_{n}$, $w_{n}$, and $u_{n}$.  3:**(Stage I: recovery)** Predict $\hat{Y}=\mathrm{Head}\;\left(T(X)\right)$ and obtain the recovered representation ${\hat{r}}_{\mathrm{clean}}$ from $\hat{Y}$.  4:**(Optional Toeplitz lifting)** Reconstruct ${\hat{R}}_{T}=\mathrm{Toeplitz}\;\left({\hat{r}}_{\mathrm{clean}},{\hat{r}}_{\mathrm{clean}}^{*}\right)$, and optionally apply diagonal loading.  5:**(Stage-II features)** Build DOA-stage features $U\in R^{N\times F}$ from the recovered representation, and standardize to obtain $\tilde{U}$.  6:**(Stage II: DOA regression)** Estimate$$\hat{\theta }=\psi \;\left(\mathrm{Pool}\;\left(T(\tilde{U})\right)\right),$$
optionally conditioned on an SNR metadata.  7:**(Training)** Optimize Stage I using $L_{\mathrm{rec}}$ and Stage II using $L_{\mathrm{doa}}$ (either sequentially: Stage I → Stage II, or jointly with weighted sum).


## 3. Experiments and Results

### 3.1. Experimental Setup

We conducted experiments on a large-scale dataset containing 200,000 samples. The input data were generated using a six-sensor sparse array with element indices 0, 1, 4, 5, 11, 13, under SNR ranging from −5 to 10 dB with 1024 snapshots per sample. The detailed model configurations and hyperparameter settings for each module are summarized in Table 1 and Table 2.

### 3.2. RMSE Performance Under Different SNR Levels

**Figure 2:**
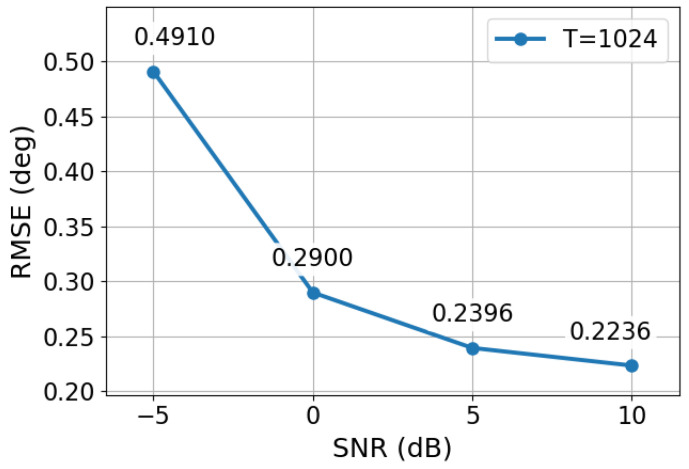
RMSE performance of the proposed method under different SNR levels.

To further validate the DOA estimation performance of the proposed model under different noise conditions, we randomly generated 100 two-source test cases at four SNR levels, namely −5, 0, 5, and 10 dB. For each test case, the two DOAs were estimated via the proposed two-stage framework, and the RMSE was computed between the estimated and true DOAs. The corresponding results are presented in Figure 2.

As shown in Figure 2, the RMSE of the proposed method decreases as the SNR increases. This trend is consistent with the fact that higher SNR leads to more reliable correlation estimates and reduces the difficulty of both correlation-vector recovery and subsequent DOA regression. In the low-SNR case of −5 dB, the received observations are strongly contaminated by noise, and the partial correlation vector obtained from the sparse array becomes less accurate. As a result, the DOA estimation error is relatively larger. Nevertheless, the proposed framework still maintains stable estimation performance, indicating that the recovery stage can effectively suppress part of the noise-induced distortion in the correlation domain.

When the SNR increases from −5 dB to 0 dB and 5 dB, the RMSE evidently decreases, demonstrating that the recovered correlation-domain representation becomes more accurate as the observation quality improves. At 10 dB, the RMSE further decreases and reaches a relatively low level. These results verify that the proposed recovery-before-inference framework can adapt to different noise conditions and provide robust DOA estimation performance over a wide SNR range.

**Figure 3:**
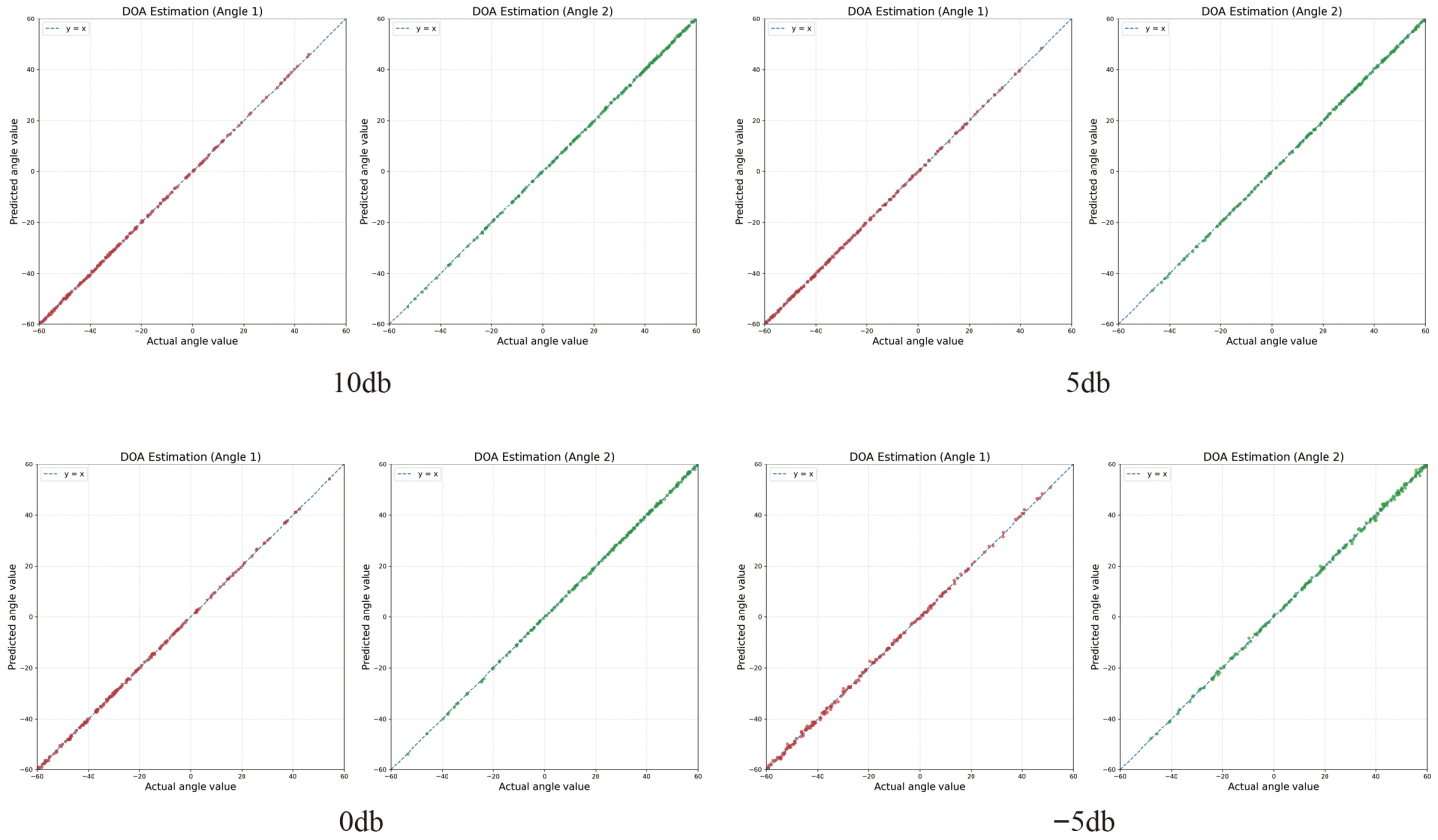
Scatter plots of the proposed method under different SNRs.

We also plot the scatter distribution of the estimated DOAs for 200 two-source samples at −5, 0, 5, and 10 dB, as shown in Figure 3.

It should be noted that this scatter plot is used only as a supplementary visualization to illustrate the consistency and dispersion of the estimated DOAs, while the quantitative conclusions are mainly supported by the RMSE and ACC results.

### 3.3. RMSE Performance Under Different Snapshot Numbers

**Figure 4:**
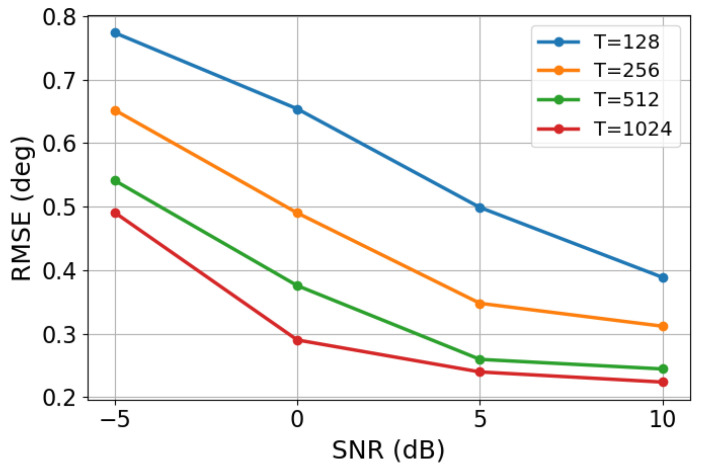
RMSE comparison for different snapshot numbers.

To assess the performance of the proposed method under different snapshot numbers, we generated 100 two-source test cases for each of four snapshot settings, namely 1024, 512, 256, and 128. For each setting, the DOAs were estimated by the proposed framework, and the corresponding RMSE values were computed. The results are shown in Figure 4.

As shown in Figure 4, the RMSE decreases as the number of snapshots increases. This trend is consistent with the statistical property that a larger number of snapshots generally leads to a more accurate estimate of the reference correlation vector, thereby facilitating both the correlation-recovery stage and the subsequent DOA regression stage. When only 128 snapshots are available, the observed data are relatively limited, and the estimated correlation information becomes more noisy and less stable, which results in a larger estimation error.

As the number of snapshots increases from 128 to 256 and 512, the RMSE decreases gradually, indicating that the proposed framework can effectively benefit from improved statistical reliability in the input observations. When 1024 snapshots are used, the estimation accuracy is further improved and reaches the best performance among the tested settings. These results demonstrate that the proposed method is applicable over a range of snapshot conditions and maintains reasonable estimation capability even in snapshot-limited scenarios, while achieving higher accuracy when more snapshots are available.

### 3.4. Comparison with Baseline Methods

**Figure 5:**
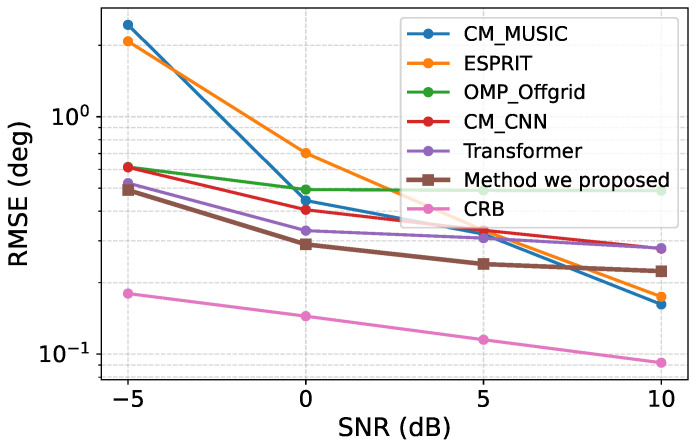
RMSE comparison of different methods under different SNR levels.

To further evaluate the effectiveness of the proposed framework, we compared it with several representative competing methods under different SNR conditions. For each SNR level, 100 two-source test cases were generated, and the corresponding RMSE values of all compared methods were computed. The comparison results are shown in Figure 5.

As shown in Figure 5, the proposed method achieves lower RMSE than the competing baselines over most of the tested SNR range. In particular, under low- and medium-SNR conditions, the advantage of the proposed method is more evident. This result indicates that the proposed recovery-before-inference framework is effective in alleviating the adverse influence of noise and incomplete sparse-array observations. By first reconstructing a clean and complete correlation-domain representation and then performing DOA regression, the proposed method provides more reliable estimation when the raw covariance information is noisy or partially missing.

It can also be observed that, in the high-SNR region, some classical methods achieve slightly lower RMSE than the proposed framework. This phenomenon is reasonable because, when the SNR is high, the sample covariance matrix becomes more accurate, and classical subspace-based estimators can better exploit their favorable asymptotic properties under well-matched signal models. Nevertheless, the proposed method still maintains competitive performance at high SNR, while showing clearer advantages in the more challenging low-SNR regime. Overall, these comparison results demonstrate that the proposed method offers a favorable trade-off between robustness and accuracy across different noise conditions.

### 3.5. Accuracy Comparison Under Different SNR Levels

**Figure 6:**
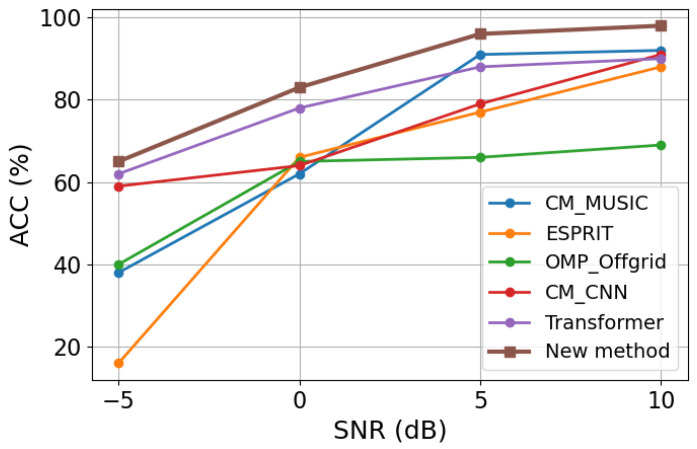
ACC comparison of different methods under different SNR levels.

To further evaluate the stability of angle estimation, we adopt the accuracy (ACC) metric in addition to RMSE. A trial is regarded as successful only when the absolute errors of both estimated DOAs are within 0.5°. Under this criterion, the ACC values of different methods are computed at different SNR levels. The corresponding results are shown in Figure 6.

As shown in Figure 6, the ACC of all compared methods generally increases with the SNR. This is consistent with the fact that higher SNR leads to more reliable observations and more accurate covariance-related information, thereby improving the probability of correctly estimating both source directions within the prescribed tolerance. At low SNR, the estimation task becomes more challenging due to stronger noise interference, and the success rate of all methods is correspondingly lower.

Compared with the competing methods, the proposed framework achieves higher ACC across the tested SNR levels. This result indicates that the proposed method not only reduces the average estimation error in terms of RMSE, but also improves the probability of obtaining fully correct two-source estimation results under a strict angular-error criterion. In particular, the advantage in ACC further demonstrates that the recovery-before-inference strategy can provide more stable DOA estimates in noisy sparse-array scenarios. The ACC curves are consistent with the RMSE results and further confirm the effectiveness and stability of the proposed method.

### 3.6. Verification of the Recovery Stage with an ESPRIT Back-End

To further verify the effectiveness of the proposed recovery stage, we additionally examined whether the reconstructed correlation vector can support downstream DOA estimation in a classical covariance-domain pipeline. In this experiment, the true DOAs were fixed at 19.44° and 24.44°, the number of snapshots was set to 1024 for each trial, and 50 Monte Carlo trials were conducted under each SNR setting. The recovered correlation vector produced by the first stage of the proposed framework was first used to construct a Hermitian Toeplitz covariance matrix by enforcing the ULA structural constraint. Based on this recovered covariance matrix, ESPRIT was then applied to estimate the DOAs.

**Figure 7:**
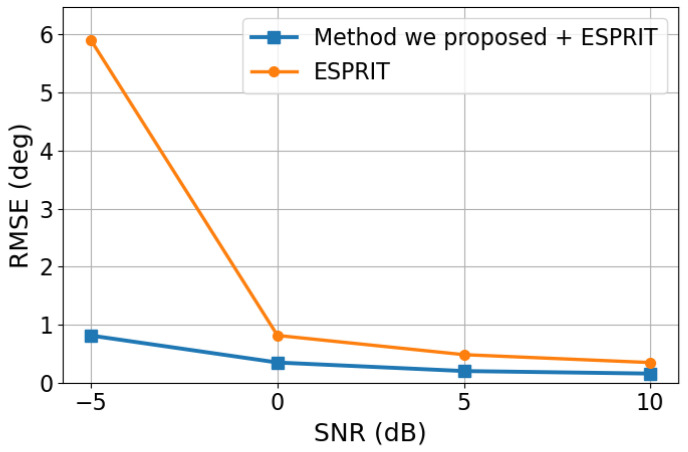
Comparison of recovery quality using an ESPRIT back-end.

For comparison, a baseline covariance matrix was also constructed using the conventional difference–coarray procedure under the same test conditions, and ESPRIT was applied in the same manner. In this way, the comparison focuses on the quality of the covariance representation provided by the proposed recovery stage and the conventional difference–coarray method. The corresponding results are shown in Figure 7.

As shown in Figure 7, the covariance matrix reconstructed from the recovered correlation vector yields lower RMSE than the covariance obtained from the conventional difference–coarray approach. The advantage is especially evident in the low-SNR regime, where the raw covariance estimate is more seriously affected by noise and incomplete observations. This result indicates that the proposed recovery stage not only benefits the end-to-end Transformer-based DOA regression, but also provides a physically meaningful and practically useful intermediate representation for classical subspace-style processing.

As the SNR increases, the performance of both approaches improves, since the underlying correlation information becomes more reliable. Nevertheless, the proposed recovery-based covariance remains more accurate over the tested SNR range, which confirms that the Stage-I output is not merely a latent neural feature, but an effective covariance-domain representation that can be directly exploited by traditional DOA estimators. Therefore, this experiment further validates the interpretability and practical utility of the proposed recovery-before-inference framework.

### 3.7. Robustness Against Array Model Errors

To further evaluate the robustness of the proposed framework against array model errors, we consider sensor-position perturbations in the test stage. In practical sparse-array systems, the actual sensor locations may slightly deviate from their nominal positions because of installation errors, calibration inaccuracies, or hardware imperfections. Such deviations introduce array–manifold mismatch and may degrade the accuracy of covariance-based DOA estimation.

Let $p_{n}$ denote the nominal position of the *n*th sensor. The perturbed sensor position is modeled as

(77) $${\tilde{p}}_{n}=p_{n}+\Delta p_{n}$$

where $\Delta p_{n}$ denotes the position perturbation. In this experiment, different perturbation levels are considered, including no perturbation, 0.002d, 0.005d, and 0.01d, where *d* is the nominal inter-element spacing. The trained model is directly evaluated on the perturbed test data without retraining, so that the robustness of the proposed method to unseen array–manifold mismatch can be examined.

**Figure 8:**
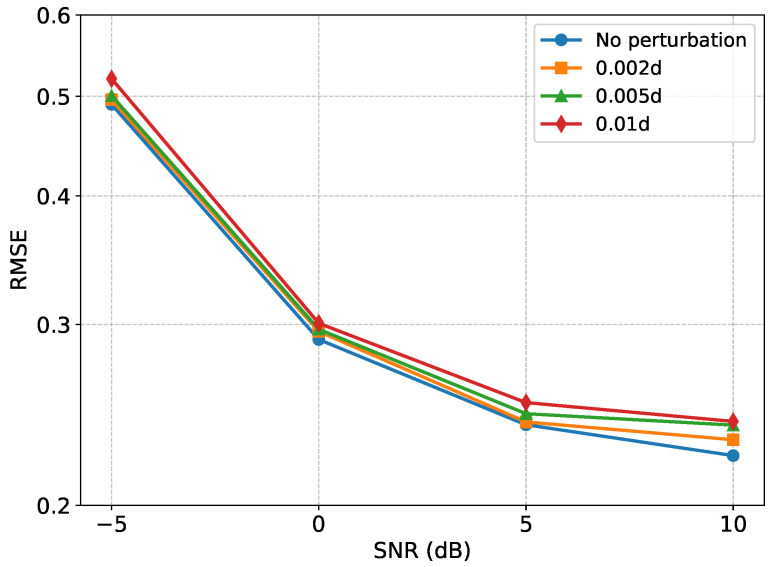
RMSE performance under sensor-position perturbations.

The corresponding RMSE results are shown in Figure 8. It can be observed that the estimation error generally increases as the perturbation level becomes larger, which is consistent with the fact that sensor-position errors distort the steering vectors and reduce the consistency between the actual and nominal array manifolds. Nevertheless, the proposed method maintains a relatively stable performance under mild perturbations. Compared with the no-perturbation case, the RMSE curves under 0.002d and 0.005d perturbations exhibit only limited degradation over the tested SNR range. Even when the perturbation level increases to 0.01d, the proposed framework still preserves the overall decreasing RMSE trend as the SNR increases.

These results indicate that the proposed two-stage framework has a certain degree of tolerance to practical array model errors. This robustness can be attributed to the correlation-recovery stage, which learns to reconstruct a denoised and complete correlation-domain representation from imperfect observations rather than relying solely on the raw perturbed covariance estimate. However, the performance degradation under larger perturbations also suggests that severe array–manifold mismatch remains challenging, and mismatch-aware training or explicit array calibration may further improve robustness in future work.

### 3.8. Performance Evaluation Under Three-Source DOA Estimation

To further evaluate the capability of the proposed framework in multiple-DOA estimation scenarios, an additional three-source experiment is conducted. In this experiment, each test sample contains three far-field sources, and the proposed framework outputs three DOA estimates simultaneously. The sparse-array configuration is kept the same as in the previous experiments, and the number of snapshots is fixed at 1024. Four SNR levels, namely −5, 0, 5, and 10 dB, are considered. For each SNR level, 100 independent test samples with randomly generated three-source DOAs are used for evaluation. The RMSE is computed after globally matching the estimated DOA set with the ground-truth DOA set.

**Figure 9:**
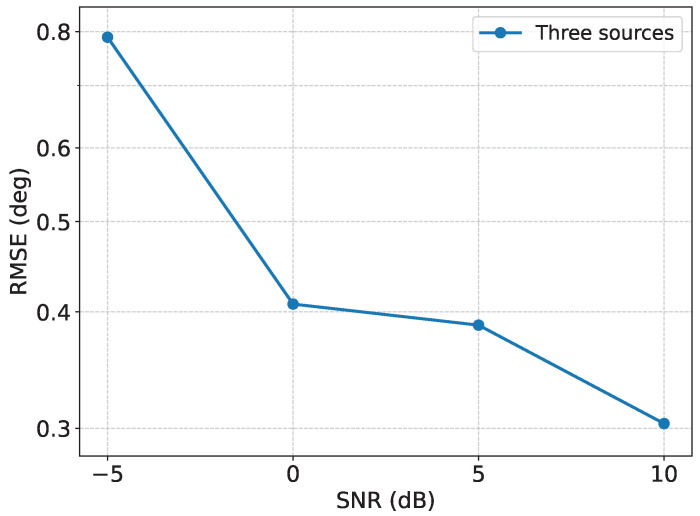
RMSE performance under three-source DOA estimation.

As shown in Figure 9, the proposed method maintains stable estimation performance in the three-source scenario, and the RMSE generally decreases as the SNR increases. Compared with the two-source case, the three-source setting is more challenging because more angular parameters need to be jointly estimated from the same sparse-array observations. Nevertheless, the proposed recovery-before-inference framework still provides reasonable estimation accuracy under different SNR levels. This result further verifies that the proposed method is not limited to the two-source case and can be applied to multiple DOA estimation scenarios.

## 4. Discussion

The results demonstrate that the proposed two-stage Transformer framework effectively mitigates the performance degradation caused by sparse sampling and limited snapshots. When only partial sensors are available, the sample covariance computed from incomplete measurements becomes biased and noisy; under small snapshot numbers, it can also be ill-conditioned, which directly weakens classical subspace-based estimators. By operating in the correlation domain and explicitly accounting for missing entries via masking, the Stage-I module produces a denoised and completed representation that is more reliable for subsequent DOA inference.

A key implication is that the recovered output can be interpreted as the first column of the covariance matrix, enabling a structured covariance reconstruction and thereby linking learning-based recovery with conventional second-order processing. This also explains why the recovered representation improves both end-to-end regression accuracy and the downstream behavior of subspace-style pipelines under challenging conditions.

It is also worth noting that the proposed method is not always superior to classical subspace estimators in high-SNR regimes. This phenomenon is reasonable from both statistical and learning-based perspectives. When the SNR is high and the number of snapshots is sufficiently large, the sample covariance matrix becomes more reliable, and the array model mismatch caused by noise is relatively weak. In this case, classical methods such as MUSIC and ESPRIT can effectively exploit the eigenspace structure of the covariance matrix and approach their favorable asymptotic performance under well-matched signal assumptions. Therefore, their performance at high SNR can be slightly better than that of the proposed learning-based method.

The proposed framework is designed mainly for challenging sparse-array scenarios where the covariance information is incomplete, noisy, or ill-conditioned due to missing sensors, low SNR, and limited snapshots. The recovery stage learns a robust mapping from partial and noisy reference correlations to a clean and complete correlation-domain representation. Such a robustness-oriented learning design improves stability in difficult regimes, but it may introduce a small data-driven bias when the test condition becomes very favorable, such as at high SNR. Therefore, the main advantage of the proposed method lies not in replacing classical estimators under ideal high-SNR conditions, but in providing a more stable and reliable solution when covariance estimation is degraded by sparse observations and adverse noise conditions.

Although the present study focuses on a ULA-based sparse-array configuration, the proposed recovery-before-inference framework is not conceptually restricted to this specific geometry. The first-stage recovery module operates on a correlation-domain representation with explicit observation masks and sensor-position information. Therefore, for other array geometries, such as nonuniform linear arrays, nested arrays, coprime arrays, or planar arrays, the sensor locations and missing-element patterns can be encoded into the input tokens, and the model can be retrained to recover a suitable correlation-domain representation under the corresponding array manifold. Similarly, the second-stage Transformer regressor can be adapted by using geometry-aware features or positional encodings associated with the target array configuration.

The Hermitian Toeplitz covariance reconstruction used in this work relies on the spatial shift-invariance property of the ULA. Therefore, this specific Toeplitz lifting step is ULA-dependent and cannot be directly applied to arbitrary array manifolds without modification. For other array geometries, the recovered representation should be mapped to an array-specific covariance structure or a manifold-consistent reconstruction form. Thus, the overall two-stage framework is extensible to other array manifolds, while the current Toeplitz covariance reconstruction should be regarded as a ULA-specific implementation.

Future work will focus on enforcing the positive semidefiniteness of the covariance matrix reconstructed from the Transformer-recovered vector. This would make the recovered covariance directly compatible with eigenvalue- and eigendecomposition-based DOA estimators, and further improve numerical stability without relying on heuristic adjustments.

## 5. Conclusions

In this paper, we present a two-stage Transformer framework for sparse-array DOA estimation. The first stage performs correlation-domain recovery, learning to reconstruct a clean and complete correlation vector from partially observed measurements, while the second stage leverages the recovered representation to predict multi-source DOA. Comprehensive experiments on a large-scale dataset and additional controlled tests demonstrate that the proposed method provides improved accuracy and stability across a wide range of SNR and snapshot numbers, with particularly pronounced gains under low-SNR and snapshot-limited conditions where covariance estimation becomes unreliable and conventional estimators are most sensitive to noise and missing sensors. At higher SNRs, classical methods remain competitive and may achieve slightly lower errors, while the proposed framework maintains comparable performance.

In addition, the effectiveness of the recovery stage is validated through a classical back-end evaluation: a structured covariance matrix constructed from the recovered correlation vector enables more reliable ESPRIT-based DOA estimation than the conventional difference–coarray processing under identical settings, with the advantage being most evident in challenging low-SNR regimes. Future work will focus on enforcing the positive semidefiniteness of the reconstructed covariance matrix, so that the recovered output becomes directly compatible with eigenvalue- and eigendecomposition-based DOA estimators and offers improved numerical stability without heuristic adjustments.

## Figures and Tables

**Table 1:** Transformer for correlation-vector recovery.

Item	Value
Token dimension	5
Attention heads	8
Dropout (encoder/input)	0.1
$d_{\mathrm{model}}$	256
Layer dropout	0.1
Recovery head	256→2
Head dropout	0

**Table 2:** Transformer regressor for DOA estimation.

Item	Value
Input feature dimension	*F*
Attention heads	8
Dropout (encoder/input)	0.1
$d_{\mathrm{model}}$	512
Layer dropout	0.1
Pooling	Attention
Pooling dropout	0
Regression head	512→512→K
Head dropout	0.1

## Data Availability

The data supporting the findings of this study are available from the corresponding author upon reasonable request.

## Abbreviations

The following abbreviations are used in this manuscript:

SNR	Signal-to-Noise Ratio
DOA	Direction of Arrival
ACC	Accuracy
MUSIC	Multiple Signal Classification
ESPRIT	Estimation of Signal Parameters via Rotational Invariance Techniques

## References

[1] Shen Q., Liu W., Cui W., Wu S. (2016). Underdetermined DOA estimation under the compressive sensing framework: A review. IEEE Access.

[2] Shmuel D.H., Merkofer J.P., Revach G., van Sloun R.J.G., Shlezinger N. (2024). SubspaceNet: Deep learning-aided subspace methods for DoA estimation. IEEE Trans. Veh. Technol..

[3] Schmidt R.O. (1986). Multiple emitter location and signal parameter estimation. IEEE Trans. Antennas Propag..

[4] Roy R., Kailath T. (1989). ESPRIT—Estimation of signal parameters via rotational invariance techniques. IEEE Trans. Acoust. Speech Signal Process..

[5] Chen Z., Chen P., Guo Z., Zhang Y., Wang X. (2023). A RIS-based vehicle DOA estimation method with integrated sensing and communication system. IEEE Trans. Intell. Transp. Syst..

[6] Zhou C., Gu Y., Shi Z., Haardt M. (2023). Structured Nyquist correlation reconstruction for DOA estimation with sparse arrays. IEEE Trans. Signal Process..

[7] Wu X., Yang X., Jia X., Tian F. (2022). A gridless DOA estimation method based on convolutional neural network with Toeplitz prior. IEEE Signal Process. Lett..

[8] Chen P., Chen Z., Liu L., Chen Y., Wang X. (2024). SDOA-Net: An efficient deep-learning-based DOA estimation network for imperfect array. IEEE Trans. Instrum. Meas..

[9] Merkofer J.P., Revach G., Shlezinger N., Routtenberg T., van Sloun R.J.G. (2023). DA-MUSIC: Data-driven DoA estimation via deep augmented MUSIC algorithm. IEEE Trans. Veh. Technol..

[10] Shmuel D.H., Merkofer J.P., Revach G., van Sloun R.J.G., Shlezinger N. (2023). Deep root MUSIC algorithm for data-driven DoA estimation. Proceedings of the IEEE International Conference on Acoustics, Speech and Signal Processing (ICASSP).

[11] Wu Y., Wakin M.B., Gerstoft P. (2023). Gridless DOA estimation with multiple frequencies. IEEE Trans. Signal Process..

[12] Zhao H., Shi Y., He W., Sun H., Wang H., Liu J., Gui L. (2025). Novel graph neural network and GNN-C-Transformer model construction for direction of arrival estimation. Digit. Signal Process..

[13] Wang X., Zhao L., Jiang Y. (2023). Super augmented nested arrays: A new sparse array for improved DOA estimation accuracy. IEEE Signal Process. Lett..

[14] Guo M., Zhang Y.D., Chen T. (2018). DOA estimation using compressed sparse array. IEEE Trans. Signal Process..

[15] Qin G., Amin M.G., Zhang Y.D. (2019). DOA estimation exploiting sparse array motions. IEEE Trans. Signal Process..

[16] Hu N., Ye Z., Xu X., Bao M. (2013). DOA estimation for sparse array via sparse signal reconstruction. IEEE Trans. Aerosp. Electron. Syst..

[17] Liu Y., Chen H., Wang B. (2021). DOA estimation based on CNN for underwater acoustic array. Appl. Acoust..

[18] Zheng S., Yang Z., Shen W., Zhang L., Zhu J., Zhao Z., Yang X. (2024). Deep learning-based DOA estimation. IEEE Trans. Cogn. Commun. Netw..

[19] Ge S., Li K., Rum S.N.B.M. (2021). Deep learning approach in DOA estimation: A systematic literature review. Mob. Inf. Syst..

[20] Huang H., Yang J., Huang H., Gui G. (2018). Deep learning for super-resolution channel estimation and DOA estimation based massive MIMO system. IEEE Trans. Veh. Technol..

[21] Wang W., Zhou L., Ye K., Sun H., Hong S. (2024). A DOA estimation method based on an improved Transformer model for uniform linear arrays with low SNR. IET Signal Process..

[22] Wu Z., Wang J., Zhou Z. (2024). Two-dimensional coherent polarization–direction-of-arrival estimation based on sequence-embedding fusion Transformer. Remote Sens..

[23] Ji J., Mao W., Xi F., Chen S. (2024). TransMUSIC: A transformer-aided subspace method for DOA estimation with low-resolution ADCs. Proceedings of the 2024 IEEE International Conference on Acoustics, Speech and Signal Processing (ICASSP).

